# The role of GITR single‐positive cells in immune homeostasis

**DOI:** 10.1002/iid3.148

**Published:** 2017-02-23

**Authors:** Giuseppe Nocentini, Luigi Cari, Graziella Migliorati, Carlo Riccardi

**Affiliations:** ^1^ Section of Pharmacology, Department of MedicineUniversity of PerugiaPerugiaItaly

**Dear Editor**:

In 2003, Uraushihara et al. demonstrated that CD4^+^CD25^−^GITR^+^ T cells express cytotoxic T lymphocyte–associated protein (CTLA‐)4, are anergic, produce interleukin (IL)‐10 and transforming growth factor (TGF)‐β, and exhibit regulatory activity 1. In 2011, we demonstrated in human peripheral blood the existence of CD4^+^CD25^low^GITR^+^ T lymphocytes isolated from a CD4^+^ population depleted of CD25^+^ cells and positively selected after GITR (TNF receptor superfamily member 18) staining 2. The cells were designated CD25^low^ (although flow cytometry suggested they were CD25^−^) to convey that they expressed higher levels of CD25 than the effector cells did. In a subsequent publication, however, these cells were designated CD4^+^CD25^low/−^GITR^+^ to avoid confusion 3. CD4^+^CD25^low/−^GITR^+^ T lymphocytes express higher levels of FOXP3 than effector cells, but much lower levels of FOXP3 than CD4^+^CD25^+^ cells (thymus‐derived T regulatory cells, tTregs). Moreover, almost all CD4^+^CD25^low/−^GITR^+^ T lymphocytes expressed the memory marker CD45RO, produced high levels of IL‐10 and TGF‐β, were anergic, and demonstrated suppressive activity even when cell‐to‐cell contact was prevented, as observed in other peripheral Treg (pTreg) subsets 2, 3, 4. Therefore, despite the slightly different characteristics of the murine and human subsets, it is reasonable to conclude that CD4^+^CD25^low/−^FOXP3^low^GITR^+^ cells are present in both mice and humans. Currently, we refer to these cells as “GITR single‐positive” (GITRsp) to emphasize the manner in which they can be sorted. As these cells express CD45RO and do not express CD25, we concluded that they are derived from activated effector T cells and are therefore pTregs, resembling type 1 Treg (Tr1) and T helper (Th)3 cells, the main pTreg subsets. However, Tr1 cells express IL‐10 and are FOXP3^−^; and Th3 cells express TGF‐β and are FOXP3^+^ 5, 6. Consequently, GITRsp cells appear to be neither Tr1 nor Th3 cells.

Some data suggest that GITRsp cells play a relevant functional role in maintaining immune homeostasis. Uraushihara et al. demonstrated that CD4^+^GITR^+^ T cells regulate the mucosal immune responses and intestinal inflammation irrespective of CD25 expression 1. In particular, CD4^+^CD25^−^GITR^+^ cells inhibit the development of classic CD4^+^CD45RB^high^ transfer colitis in severe combined immunodeficiency mice. Ono et al. compared the effect of transferred GITR‐ and CD25‐depleted T cells in nude mice 7. Ninety percent of mice that received GITR‐depleted cells died due to autoimmune diseases, whereas no mice that received CD25‐depleted cells died, suggesting the presence of a GITR^+^CD25^−^ Treg population that is crucial for maintaining immune homeostasis.

Our group observed a potential homeostatic role of GITRsp cells in humans. In fact, we demonstrated the expansion of the GITRsp Treg subpopulation in the peripheral blood of patients with inactive Sjögren's syndrome, but not in patients with active disease 4. A similar result was observed in patients with systemic lupus erythematosus (SLE). In patients with SLE with a SLE Disease Activity Index (SLEDAI) score of 0, the mean proportion of GITRsp Tregs among CD4^+^ cells was >4%, whereas it was about 1% in patients with SLEDAI scores>0 (i.e., active disease), similar to the percentage of GITRsp Tregs in healthy controls 3. Interestingly, the attempt to use CD25 as a predictive marker in patients with SLE was unsuccessful 3. Therefore, it is reasonable to assume that GITRsp Treg expansion impedes disease development and that a treatment favoring GITRsp Treg expansion could be curative 8, 9.

A recently published paper by Yoshida et al. in this journal demonstrates that GITRsp Treg expansion slowed disease progression and prevented relapse in a murine model of arthritis 10. Mice showing disease signs 9–10 days after glucose‐6‐phosphate isomerase (GPI) peptide treatment were co‐treated with GPI and fingolimod for 5 days. The combined treatment not only decreased the arthritis clinical score during treatment, it also prevented relapse upon re‐treatment with GPI on day 32 (resensitization). Mice treated with fingolimod alone or GPI alone had clinical scores similar to placebo‐treated mice and were sensitive to resensitization. As no differences in self‐antibody concentrations were observed among the four groups, the authors hypothesized that the combined treatment altered the balance between effector T cells and Tregs in the combined treatment group as compared to the fingolimod‐treated group. The authors did not find any difference in the percentage of FOXP3^+^ cells (e.g., tTregs and Th3 cells) in the inguinal lymph nodes of the mice. On the contrary, they found that CD4^+^CD25^−^FOXP3^−^GITR^+^ cells (i.e., GITRsp) had increased. Moreover, they demonstrated that GITRsp Tregs express high levels of CTLA‐4 similar to that expressed by CD4^+^CD25^+^FOXP3^+^GITR^+^ tTregs, produce much more IL‐10 than CD4^+^CD25^+^FOXP3^+^GITR^+^ tTregs, and have regulatory activity. Therefore, the findings of Yoshida et al. further illustrate the role GITRsp cells might play in decreasing T cell activation/proliferation and controlling autoimmune diseases. Indeed, we demonstrated that the number of activated CD4^+^ T cells (CD4^+^CD25^+^GITR^−^) was inversely correlated with the number of GITRsp cells in patients with SLE 3.

Although fingolimod promoted permanence of activated T cells in the lymph nodes in the experimental model used by Yoshida et al., it induced the expansion of GITRsp cells only in association with antigenic stimulation. We may hypothesize that the fingolimod‐dependent increase in T cell density in the lymph nodes favors GITR triggering by the membrane‐bound GITR ligand (GITRL) expressed by activated T cells and antigen‐presenting cells (APC) and that increased GITR triggering favors the expansion and/or differentiation of activated/antigen‐specific GITRsp cells. Indeed, the expansion of GITRsp cells has been described in GITRL transgenic mice 11.

In summary, Yoshida et al. suggest that increased GITRsp Tregs can prevent relapse following resensitization, supporting the hypothesis that drug‐induced expansion of GITRsp Tregs is a viable treatment strategy for patients with autoimmune diseases and that GITRsp pTregs can be used in cell‐based therapeutic approaches 8, 12. Several studies in murine models have demonstrated that pTreg infusion is more effective than tTreg infusion in the treatment of autoimmune diseases. For example, Th3 pTregs suppress lupus‐like chronic graft‐versus‐host disease (GVHD), and the adoptive transfer of antigen‐specific Th3 pTregs before the induction of collagen‐induced arthritis (CIA) decreases the incidence of CIA 13, 14. In that model, the CIA score was decreased when the infusion was performed 14 days after CIA induction, while no improvement was observed after tTreg infusion. Indeed, pTregs maintain their regulatory activity in an inflammatory microenvironment while tTregs do not 15. Some studies on both murine and human tumor‐infiltrating lymphocytes (TIL) have suggested that GITRsp cells are activated tissue‐ and antigen‐specific pTregs 9. For example, GITR expression in Tregs residing in melanoma was approximately 10‐fold higher than that of Tregs in the spleens of the same animals 16. In patients with head and neck squamous cell carcinoma, TIL Tregs producing IL‐10/TGF‐β are best detected by GITR, which is expressed in 83% of TIL Tregs and in about 5% of peripheral blood mononuclear cell (PBMC) Tregs 17. For these reasons, GITRsp pTregs are candidate cell‐based therapeutic approaches for treating autoimmune diseases 8, 12.

Before GITRsp pTregs are tested in humans, however, several issues should be addressed. For example, it is crucial to determine whether GITRsp Tregs have a stable phenotype and whether they continue to expand over time. Our observation of disease inactivity in patients with SLE and the 17‐day interval between the end of GPI/fingolimod co‐treatment and resensitization suggests a certain phenotype stability, but future studies should address this question better.

## Disclosure statement

Giuseppe Nocentini was Amgen Consultant in 2014 concerning GITR modulators in disease treatments and was supported by the Ministero dell'Istruzione dell'Università e della Ricerca (MIUR) for the “Modulation of T lymphocyte activity in autoimmune diseases” project (2008SKTMME_003). Carlo Riccardi was supported by MIUR for the “GITR‐derived signals in T lymphocyte regulation” project (2006052432_001) and the “Identification of new pharmacological targets for treating allodynia and peripheral and central immune‐inflammation” project. He has also received financing from the Associazione Italiana per la Ricerca contro il Cancro (AIRC) (IG‐10677 and IG‐14291). The authors have no other relevant affiliation or financial involvement with any organization or entity with a financial interest in or financial conflict with the subject matter or materials discussed in the manuscript.

## References

[1] Uraushihara, K. , T. Kanai , K. Ko , T. Totsuka , S. Makita , R. Iiyama , T. Nakamura , and M. Watanabe . 2003 Regulation of murine inflammatory bowel disease by CD25+ and CD25‐ CD4+ glucocorticoid‐induced TNF receptor family‐related gene+ regulatory T cells. J. Immunol. 171:708–716. 12847237 10.4049/jimmunol.171.2.708

[2] Bianchini, R. , O. Bistoni , A. Alunno , M. G. Petrillo , S. Ronchetti , P. Sportoletti , E. B. Bocci , G. Nocentini , R. Gerli , and C. Riccardi . 2011 CD4(+) CD25(low) GITR(+) cells: a novel human CD4(+) T‐cell population with regulatory activity. Eur. J. Immunol. 41:2269–2278. 21557210 10.1002/eji.201040943

[3] Nocentini, G. , A. Alunno , M. G. Petrillo , O. Bistoni , E. Bartoloni , S. Caterbi , S. Ronchetti , G. Migliorati , C. Riccardi , and R. Gerli . 2014 Expansion of regulatory GITR+CD25 low/‐CD4+ T cells in systemic lupus erythematosus patients. Arthritis Res. Ther. 16:444. 25256257 10.1186/s13075-014-0444-x PMC4209023

[4] Alunno, A. , M. G. Petrillo , G. Nocentini , O. Bistoni , E. Bartoloni , S. Caterbi , R. Bianchini , C. Baldini , I. Nicoletti , C. Riccardi , et al. 2013 Characterization of a new regulatory CD4+ T cell subset in primary Sjogren's syndrome. Rheumatology (Oxford) 52:1387–1396. 23674818 10.1093/rheumatology/ket179

[5] Roncarolo, M. G. , S. Gregori , R. Bacchetta , and M. Battaglia . 2014 Tr1 cells and the counter‐regulation of immunity: natural mechanisms and therapeutic applications. Curr. Top. Microbiol. Immunol. 380:39–68. 25004813 10.1007/978-3-662-43492-5_3

[6] Carrier, Y. , J. Yuan , V. K. Kuchroo , and H. L. Weiner . 2007 Th3 cells in peripheral tolerance. I. Induction of Foxp3‐positive regulatory T cells by Th3 cells derived from TGF‐beta T cell‐transgenic mice. J. Immunol. 178:179–185. 17182553 10.4049/jimmunol.178.1.179

[7] Ono, M. , J. Shimizu , Y. Miyachi , and S. Sakaguchi . 2006 Control of autoimmune myocarditis and multiorgan inflammation by glucocorticoid‐induced TNF receptor family‐related protein(high), Foxp3‐expressing CD25+ and CD25‐ regulatory T cells. J. Immunol. 176:4748–4756. 16585568 10.4049/jimmunol.176.8.4748

[8] Petrillo, M. G. , S. Ronchetti , E. Ricci , A. Alunno , R. Gerli , G. Nocentini , and C. Riccardi . 2015 GITR+ regulatory T cells in the treatment of autoimmune diseases. Autoimmun. Rev. 14:117–126. 25449679 10.1016/j.autrev.2014.10.011

[9] Ronchetti, S. , E. Ricci , M. G. Petrillo , L. Cari , G. Migliorati , G. Nocentini , and C. Riccardi . 2015 Glucocorticoid‐induced tumour necrosis factor receptor‐related protein: a key marker of functional regulatory T cells. J. Immunol. Res. 2015:171520. 25961057 10.1155/2015/171520 PMC4413981

[10] Yoshida, Y. , N. Mikami , Y. Matsushima , M. Miyawaki , H. Endo , R. Banno , T. Tsuji , T. Fujita , and T. Kohno . 2016 Combination treatment with fingolimod and a pathogenic antigen prevents relapse of glucose‐6‐phosphate isomerase peptide‐induced arthritis. Immun. Inflamm. Dis. 4:263–273. 27621810 10.1002/iid3.111 PMC5004282

[11] Carrier, Y. , M. J. Whitters , J. S. Miyashiro , T. P. LaBranche , H. E. Ramon , S. E. Benoit , M. S. Ryan , S. P. Keegan , H. Guay , J. Douhan , et al. 2012 Enhanced GITR/GITRL interactions augment IL‐27 expression and induce IL‐10‐producing Tr‐1 like cells. Eur. J. Immunol. 42:1393–1404. 22678896 10.1002/eji.201142162

[12] Nocentini, G. , G. Migliorati , and C. Riccardi . 2015 Are we able to harness the immunomodulatory power of cytokines for novel autoimmune disease treatments? Am. J. Pharmacol. Toxicol. 10:37–39.

[13] Lan, Q. , X. Zhou , H. Fan , M. Chen , J. Wang , B. Ryffel , D. Brand , R. Ramalingam , P. R. Kiela , D. A. Horwitz , et al. 2012 Polyclonal CD4+Foxp3+ Treg cells induce TGFbeta‐dependent tolerogenic dendritic cells that suppress the murine lupus‐like syndrome. J. Mol. Cell. Biol. 4:409–419. 22773728 10.1093/jmcb/mjs040 PMC3523557

[14] Kong, N. , Q. Lan , M. Chen , J. Wang , W. Shi , D. A. Horwitz , V. Quesniaux , B. Ryffel , Z. Liu , D. Brand , et al. 2012 Antigen‐specific transforming growth factor beta‐induced Treg cells, but not natural Treg cells, ameliorate autoimmune arthritis in mice by shifting the Th17/Treg cell balance from Th17 predominance to Treg cell predominance. Arthritis. Rheum. 64:2548–2558. 22605463 10.1002/art.34513 PMC4364395

[15] Xu, L. , A. Kitani , I. Fuss , and W. Strober . 2007 Cutting edge: regulatory T cells induce CD4+CD25‐Foxp3‐ T cells or are self‐induced to become Th17 cells in the absence of exogenous TGF‐beta. J. Immunol. 178:6725–6729. 17513718 10.4049/jimmunol.178.11.6725

[16] Sacchetti, C. , N. Rapini , A. Magrini , E. Cirelli , S. Bellucci , M. Mattei , N. Rosato , N. Bottini , and M. Bottini . 2013 In vivo targeting of intratumor regulatory T cells using PEG‐modified single‐walled carbon nanotubes. Bioconjug. Chem. 24:852–858. 23682992 10.1021/bc400070q

[17] Strauss, L. , C. Bergmann , M. Szczepanski , W. Gooding , J. T. Johnson , and T. L. Whiteside . 2007 A unique subset of CD4+CD25highFoxp3+ T cells secreting interleukin‐10 and transforming growth factor‐beta1 mediates suppression in the tumor microenvironment. Clin. Cancer Res. 13:4345–4354. 17671115 10.1158/1078-0432.CCR-07-0472

